# Pain as the first manifestation of an acute ischemic parietal stroke: A case report

**Published:** 2020-01-05

**Authors:** Miguel Angel Saucedo, Laura De Francesco, Anibal Chertcoff, Lucrecia Bandeo, Luciana Leon Cejas, Manuel Maria Fernandez Pardal, Ariel Miquelini, Ricardo Reisin, Pablo Bonardo

**Affiliations:** 1 Department of Neurology, Hospital Britanico de Buenos Aires, Buenos Aires, Argentina; 2 Department of Radiology, Hospital Britanico de Buenos Aires, Buenos Aires, Argentina

**Keywords:** Pain, Stroke, Acute, Parietal Lobe

Sudden-onset neurological deficit (paresis, numbness, aphasia, etc.) is the most common form of presentation of ischemic stroke, although sometimes it can manifest with positive symptoms or signs such as limb-shaking transient ischemic attack. Neuropathic central pain as the first manifestation of an acute stroke is rare.^1^ Central pain has been traditionally classified according to the location of the lesion in infra-thalamic, thalamic, or supra-thalamic, however. Different studies have shown that stimulation on the superior portion of the primary somatosensory cortex of the parietal lobe, the pre- and post-rolandic sulci and the parietal operculum can trigger pain in the contralateral hemibody.^2^

We present a patient with acute cerebral infarction in the parietal cortex who presented with contralateral limb pain as the form of presentation of an acute ischemic stroke.

An 82-year-old woman was admitted to our hospital due to sudden-onset severe pain in the left lower limb. She had a history of diabetes mellitus, hypertension, dyslipidemia, hypothyroidism, right saphenectomy, dilated cardiomyopathy, and mild cognitive impairment. Sharp pain, rated using the numerical rated scale as 10/10, and located on the dorsum of the left foot. The patient had been assisted at home during pain-onset, and was transferred to the emergency department. At admission, pain had improved to 6/10 grade, but she presented a left distal left leg weakness rated with the Medical Research Council Manual Muscle Testing scale as 4/5. On examination, pedal and posterior tibial pulses were palpable and symmetrical rated as 3+ grade. Pinprick, light touch, and temperature sensation were decreased on her left leg as same as her leg foot. Lasegue's sign was absent. Vibratory sensation was diminished on her left food, but position sense was normal.

There was no sensory extinction nor ankle clonus or spasticity. Plantar responses were flexor bilaterally. Laboratory tests were done; Hemoglobin: 12.5 g/dl, white blood cells count: 8000/ul, platelet count: 250.000/ul, serum glucose: 119 mg/dl, and erythrocyte sedimentation rate (ESR): 25. 

A Doppler ultrasound of the lower limbs was performed but was unremarkable. Under the clinical suspicion of an ischemic stroke, a brain magnetic resonance imaging (MRI) was performed showing a small acute ischemic lesion on the right parasagittal parietal cortex (Figure 1). Transesophageal echocardiography revealed a grade IV aortic arch atheroma and a severe dilatation of the left atrium. Her 48-hour Holter electrocardiogram (ECG) was unremarkable as well as the MR-angiography. Somatosensory evoked potentials of the lower limbs exhibited severe involvement of sensory afferent pathways at cortical arrival bilaterally. Quantitative sensory testing of the lower limbs showed alteration of smalls sensory fibers on both feet. The patient was treated with aspirin 100 mg daily and statins; she had a good functional outcome recovery to modified Rankin Scale (mRS): 0 and National Institutes of Health Stroke Scale (NIHSS): 0. The etiology of the stroke was classified as embolic stroke of undetermined source.

**Figure 1:**
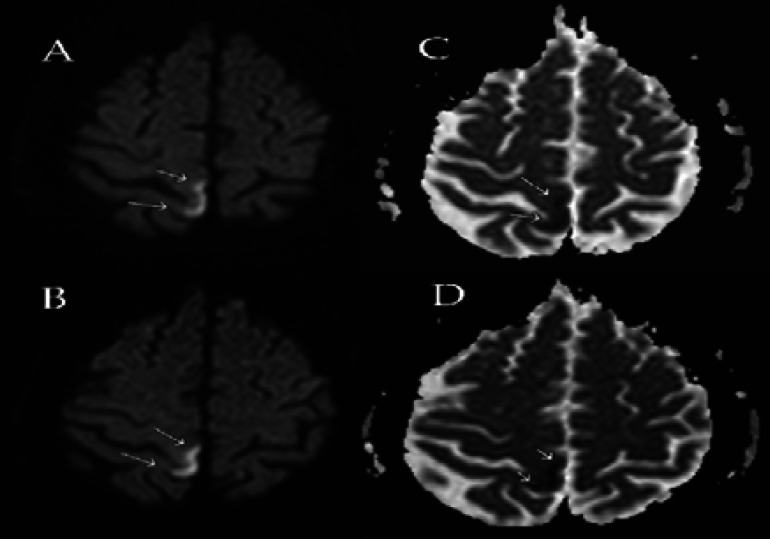
Brain magnetic resonance imaging (MRI) showing a right cortical parietal lesion hyperintense in diffusion weighted imaging sequence (A and B) and hypointense in apparent diffusion coefficient (ADC) mapping (C and D) compatible with acute ischemic stroke

Our patient presented with sudden-onset sharp pain in the left foot associated as the first manifestation of an acute ischemic stroke. Peripheral vascular pathology is the main cause of sudden-onset pain and motor deficit. However, our patient had palpable peripheral pulses in both lower limbs and a normal Doppler ultrasound study.

The occurrence of pain due to cerebral infarction is infrequent and in most cases begins after a variable period. Post-stroke pain presents in 8% to 10% of patients with acute stroke. 

Some reports related to central pain are from lesions of the thalamic region.^3^ However, pain secondary to a cortical lesion is a rare event,^4^ particularly as an initial manifestation of stroke, with only few reports in the literature; Garcia-Larrea et al. published a series of 22 patients who presented lesions in subcortical and cortical areas of the insular operculum without thalamic affectation. The cause of injury in those cases was a vascular lesion, in none of them pain was the presenting symptom of the stroke, and as opposed to our patient, stroke did not involve solely the cortex.^5^ Rossetti et al. reported a patient who presented burning pain deep in the distal left arm, as the initial symptom of stroke in association with hemicorea and hemiballismus on his left hemibody. MRI showed a cortical parietal ischemic stroke.^6^ Unlike our patient that presented with pain and subsequent motor deficit involving only the left leg, the symptoms in this case involved the whole hemibody, and did not suggest a peripheral origin.

The pathophysiological mechanisms underlying the development of pain due to central lesions and particularly due to cortical involvement does not fully understand. Some authors suggest that could be related to a disconnection between the parietal cortex and the deeper brain structures secondary to the acute injury of the primary somatosensory region that produces a decrease in the descending antinociception pathways of the somatosensory cortex that generate a reduction on the firing of "on" cells linked to 5-hydroxytryptamine-1A (5-HT1A). Others authors suggest that an increase in glutamate transport (Vglut2) on the ischemic area as well as microglial activation in the somatosensory cortex can increase neuronal excitability leading to pain perception. 

In conclusion, pain should be considered as an atypical symptom of stroke that can lead to confusion and delayed diagnosis and initiation of reperfusion therapies in these patients.

## Notes:

**How to cite this article:** Saucedo A, Francesco LD, Chertcoff A, Bandeo L, Cejas LL, Fernandez Pardal MM, et al. Pain as the first manifestation of an acute ischemic parietal stroke: A case report. Curr J Neurol 2020; 19(1): 40-2.
